# Caveolin and NOS in the Development of Muscular Dystrophy

**DOI:** 10.3390/ijms25168771

**Published:** 2024-08-12

**Authors:** Moeka Nakashima, Naoko Suga, Sayuri Yoshikawa, Satoru Matsuda

**Affiliations:** Department of Food Science and Nutrition, Nara Women’s University, Kita-Uoya Nishimachi, Nara 630-8506, Japan

**Keywords:** caveolae, caveolin, NOS, Duchenne muscular dystrophy, limb–girdle muscular dystrophy, gut microbiota, probiotics

## Abstract

Caveolin is a structural protein within caveolae that may be involved in transmembrane molecular transport and/or various intercellular interactions within cells. Specific mutations of caveolin-3 in muscle fibers are well known to cause limb–girdle muscular dystrophy. Altered expression of caveolin-3 has also been detected in Duchenne muscular dystrophy, which may be a part of the pathological process leading to muscle weakness. Interestingly, it has been shown that the renovation of nitric oxide synthase (NOS) in sarcolemma with muscular dystrophy could improve muscle health, suggesting that NOS may be involved in the pathology of muscular dystrophy. Here, we summarize the notable function of caveolin and/or NOS in skeletal muscle fibers and discuss their involvement in the pathology as well as possible tactics for the innovative treatment of muscular dystrophies.

## 1. Introduction

**Figure 1:**
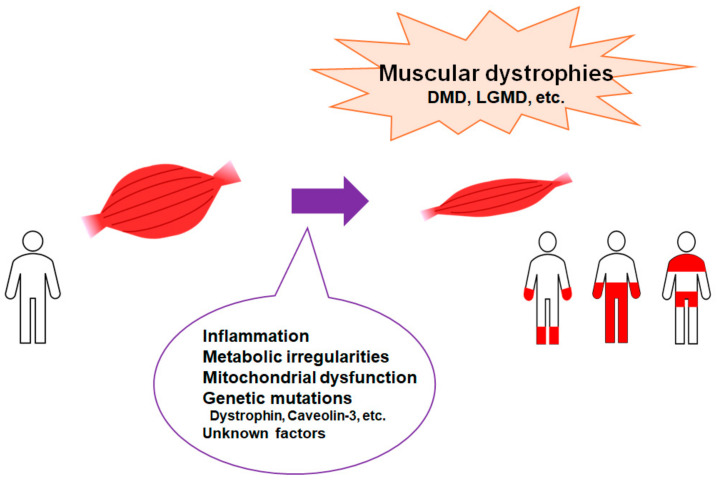
Representation of the critical factors for the development of muscular dystrophies. Muscular dystrophies are a group of illnesses that trigger the progressive loss of muscle mass. Several genetic mutations are well known for interfering with the production of molecules required to develop healthy muscle. There are various types of muscular dystrophy. Abbreviations: DMD—Duchenne muscular dystrophy; LGMD: limb–girdle muscular dystrophy.

**Figure 2:**
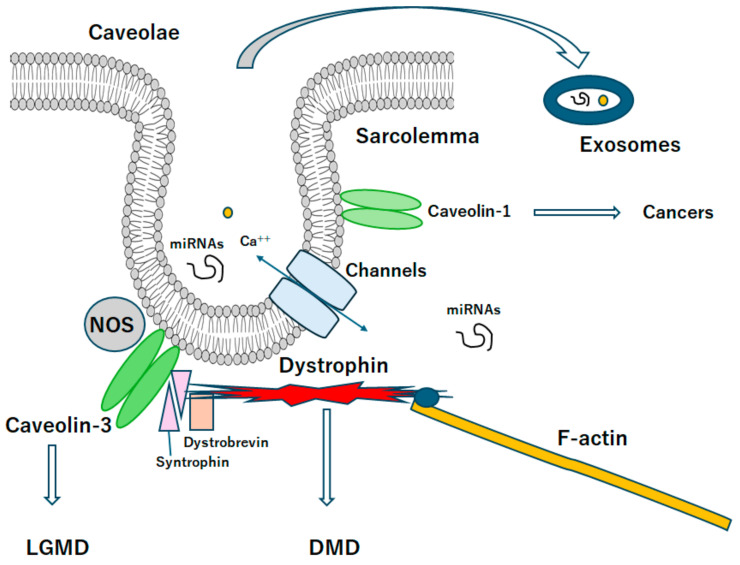
A diagrammatic representation of caveolae associated with caveolin proteins. Caveolae may be a kind of platform for various signaling and/or even exosomes, which may be related to the scaffold domain of caveolins. Note that some critical pathways for the development of various diseases have been omitted for clarity. Abbreviation, DMD: Duchenne muscular dystrophy, LGMD: limb-girdle muscular dystrophy.

Muscular dystrophies represent a group of disorders characterized by primary skeletal muscle deterioration and/or the subsequent emergence of co-morbidities such as inflammation, mitochondrial dysfunction, and metabolic irregularities. These conditions may predominantly result from mutations in certain gene products that connect the cytoskeleton to the basal lamina of muscle fibers [1] (Figure 1 and Figure 2). Duchenne muscular dystrophy (DMD) is the most common inherited X-linked form of muscular dystrophy, causing a severe and progressive neuromuscular disorder characterized by the insufficient production of dystrophin due to specific mutations in the dystrophin gene [2]. In patients with Duchenne muscular dystrophy, the X-linked mutation can interfere with the ability to produce functional dystrophin in the muscles [3]. Duchenne muscular dystrophy may end in respiratory failure and premature death [4]. Although this disorder is primarily known to affect skeletal muscle, cardiac complications such as left ventricular dysfunction are another common manifestation of Duchenne muscular dystrophy. The functional deficiency of dystrophin may lead to plasma membrane instability, causing myofiber necrosis and/or muscle weakness [5], which also results in muscle fiber inflammation, an impaired regeneration of muscle fibers, and replacement of muscle by fibrotic and adipose tissue, leading to advanced deterioration of muscle function [6,7]. Therefore, this disease manifests through compromised membrane integrity, chronic inflammation, and fibrosis as well as impaired tissue remodeling [8]. A previous study showed that lipid metabolism might be a critical metabolic disturbance in mdx mice (a genetic model of Duchenne muscular dystrophy) and uncovered the dysregulation of cholesterol and fatty acid metabolism transcription factors and the accumulation of cholesterol in dystrophic muscles [9,10]. In addition, xanthine oxidase activity has been a contributing factor in mdx mice [11]. Most of the dysregulated metabolites have a connection to energy and phospholipid metabolism, revealing the intricate metabolic remodeling of phospholipids and energy metabolism in Duchenne muscular dystrophy. Furthermore, another study has revealed the muscle metabolic remodeling patterns of Duchenne muscular dystrophy by using high-resolution mass spectrometry [12].

Although progress has been made, fundamental pathological mechanisms are still worthy of further study in order to discover beneficial therapeutic targets for Duchenne muscular dystrophy. There is no cure for Duchenne muscular dystrophy at present. Metabolic insufficiencies may have a further promotional effect on the disease progression [13]. Calcium channel blockers to restore calcium homeostasis and/or anabolic steroids to restore muscle mass may not have any clinical benefits associated with the overall development of the disease [6,14]. Glucocorticoids are currently the only medications recognized to benefit patients with Duchenne muscular dystrophy. Although they have significant side effects [15,16], they frequently prolong walking ability [17]. In addition, this treatment may be associated with various complications [18]. Compared with monotherapy, a reduction in motor function decline could be detected among the stable subgroup of patients treated with combination therapy using citrulline and/or metformin [19]. Interestingly, some studies have correlated the intestinal microbiota with the lifetime cardiovascular risk of muscular dystrophy [20]. These findings underline the urgent need to identify strategies targeting the gut microbial system and conduct in-depth analyses of the functional relationship between food and microbiota composition to modulate various human diseases, including Duchenne muscular dystrophy. These efforts may contribute to the elucidation of potential therapeutic targets and/or biomarkers.

## 2. Inflammation and Redox System May Be Involved in the Regulation of Muscular Dystrophy

Inflammation and oxidative stress may be involved in the development of muscular dystrophy. For instance, some anti-oxidants have shown to successfully reduce muscle damage in an animal model for Duchenne muscular dystrophy [21]. In addition, inflammatory markers and specific lymphocyte subsets have been identified in the blood/muscles of Duchenne muscular dystrophy patients. Additionally, T lymphocytes from the murine model of Duchenne muscular dystrophy (mdx mice) could induce muscular damage when injected into healthy murine muscle [22]. Following the muscular damage, the inflammatory phenotype may be started by damage-associated molecular arrangements that activate neutrophils by specific membrane markers such as the Toll-like receptor. Recruited pro-inflammatory macrophages may induce muscle lysis, supplying inducible nitric oxide synthase (iNOS). In dystrophic mdx mice, an excess population of this macrophage could lead to further tissue destruction with the employment of CD4/CD8+ T-cells [23]. In these ways, the pathological manifestation of Duchenne muscular dystrophy is complicatedly linked to inflammation and/or oxidative stress involvement [24]. A loss of dystrophin leads to a membrane fragility of muscle fibers, resulting in chronic inflammation, myofiber death, and/or regeneration. Eventually, functional muscle fibers might be replaced by fibrous features and/or fat [25,26]. Interestingly, the restoration of nitric oxide synthase (NOS) to the sarcolemma would be supposed to improve muscle health in Duchenne muscular dystrophy. In addition, mislocated nNOS could produce dysregulated ROS, which might contribute to the pathology of Duchenne muscular dystrophy [25,26]. Generally, NOS is transcriptionally upregulated by cytokines as well as by hypoxia. Dystrophin-deficient muscles may exhibit reductions in the expression of NOS, suggesting that NO deficiency could affect the pathology of Duchenne muscular dystrophy [27]. NO is a kind of signaling molecule with complexed and/or controversial effects. Occasionally, NOS could facilitate several cellular processes imperative for various cellular functions [28]. Remarkably, NO is usually considered to be cardioprotective [29]. Therefore, different NOS function may contribute to diverse cardiovascular diseases including high blood pressure and/or heart failure [30]. In the pathology of Duchenne muscular dystrophy, the increasing of oxidative stress may depend on an over-expression of inflammatory cells, leading to an upregulation of NOS [31]. The accumulation of inflammatory molecules could also affect the function of proteins and lipids [32,33]. Likewise, the neutrophils cause damages to muscular tissues [34]. Additionally, the oxidation of thiol (-SH) groups of cysteine residues is associated with the development of necrosis and/or fatty tissue [35]. Dystrophin can connect to intricate transmembrane proteins, including dystroglycan as well as key signaling molecules including NOS in sarcolemma [36]. In particular, NOS activities might be coupled to oxygen concentration. Therefore, mislocalized NOS could trigger abnormal protein nitrosylation and ROS, which are believed to contribute to the pathology of Duchenne muscular dystrophy. In skeletal muscle, NOS may be associate with caveolin-3. It has been suggested that muscle regeneration may be mediated by the upregulation of endothelial NOS activity and increased expression of VEGF and/or adhesion molecules [37]. Interestingly, female-specific factors including sex hormones may also play a role in those alterations for crucial aspects of Duchenne muscular dystrophy pathology [38]. Additionally, the neuronal isoform of NOS homeostasis is tightly associated with dystrophin [39]. Furthermore, the NOS is almost absent in the skeletal muscle of Duchenne muscular dystrophy patients [40]. Deficiency in NOS-derived NO might exacerbate the phenotype of Duchenne muscular dystrophy by impairing skeletal muscle function.

## 3. Relationship between Caveolin and Muscular Dystrophy

In general, NOS-derived nitric oxide (NO) could regulate a wide range of cellular functions including inflammation, apoptosis, permeability and/or cell growth. The NOS may be localized mainly near specific intracellular membrane domains including the cytoplasmic face of the Golgi apparatus and plasma membrane caveolae [41]. Flask-shaped invaginations caveolae are specialized lipid domains of the plasma membrane containing caveolins, which are key structural components and serve as a scaffold for signaling proteins [42]. In addition, signaling events in caveolae have key impact on the NOS and endothelial cell phenotypes to human health and diseases [43,44] (Figure 2). The integral membrane protein caveolin-1 (CAV1) is a major structural component of caveolae and is required in place of caveolae formation within non-muscle cells [45]. The association of CAV1 into oligomeric protein complexes is an indispensable step in caveolae biogenesis, and faults in the oligomerization could initiate various diseases [46]. In mammals, CAV1 and/or caveolae are widely distributed in many tissues where they operate for lipid homeostasis, endocytosis, and signal transduction [47]. On the other hand, the dysregulation of caveolae may contribute to the development/progression of several diseases including cancers, inflammatory diseases, hypertension, asthma, and lipodystrophy [48]. Interestingly, the P132L mutation of wild type CAV1 has been identified in several different patient samples including breast cancer and/or lung adenocarcinomas [49]. In addition to the P132L mutation, a variety of other pathogenic mutations in caveolin homologs have been identified in humans [50]. The P132L mutation has also been reported to disturb the ability of CAV1 to correctly move to the plasma membrane, suggesting it might function as a dominant negative with the wild-type CAV1 [51]. These findings emphasize the importance of P132L mutation in caveolin, which is also part of the development/progression of health and disease. Additionally, the P132L mutation is also a powerful tool to investigate how defects in trafficking and/or the oligomerization of caveolins could impede the caveolae formation [52]. Some protein mutations that could lead to muscular dystrophy may often generate deficiencies in cytoskeletal support of the muscle sarcolemma. In particular, caveolae are also cholesterol-rich microdomains that could construct mechanically deformable invaginations of the sarcolemma. For example, it is well known that mutations to caveolin-3, the main scaffolding protein of caveolae in muscle, can trigger limb–girdle muscular dystrophy (LGMD). An equivalent mutation in caveolin-3 (CAV3), P105L (curiously denoted as P104L in many studies), could show the phenotype of muscular dystrophies both in humans and/or animal models [53]. Therefore, the most equivalent to P132L may be the P105L mutation of CAV3 frequently associated with LGMD [54,55]. As for the dominant negative function, muscle biopsies from patients harboring the P105L mutation of CAV3 with an autosomal form of LGMD may detect noticeably decreased CAV3 levels compared to normal controls [55]. In addition, oligomerization and/or trafficking defects have been also observed for the P105L mutants of CAV3 [56]. Resembling the P132L mutation of CAV1, the P105L mutant protein of CAV3 can intracellularly entrap the wild-type CAV3 in mammalian heterologous expression systems [57]. However, the fine character of the oligomerization defects appears to be slightly different, as the P105L mutation of CAV3 may incline to form larger oligomers rather than that of wild-type CAV3 [56,58]. It seems possible that the P105L mutation of CAV3 may play a comparable role in destabilizing the structure of CAV3 complexes, as is the situation for CAV1 [59]. Standard expression levels of CAV3 may be protective for the sarcolemma, while the upregulation of CAV3 may be detected in several muscular dystrophies owing to the probable compensation for additional functional deficiencies [60].

## 4. MicroRNAs Might Be Involved in the Pathogenesis of Muscular Dystrophies

MicroRNAs are a class of noncoding RNAs, which are usually single-stranded RNAs that work as gene regulators on the post-transcriptional level. It has been described that the upregulation of several microRNAs in the atrial myocardium of patients may lead to the translational suppression of dystrophin and enhance the decay of the mRNA of *NOS*, which may contribute to the atrial remodeling that can promote the exacerbation of cardiac atrial arrhythmia [61]. Consistent with this finding, *NOS1* knockout mice may exhibit atrial characteristic features of the remodeling as well as an increased tendency to develop the arrhythmia [61]. However, the occurrence of cardiac arrhythmia in Duchenne muscular dystrophy patients may be comparatively low after the development of cardiomyopathy [62]. This is probably because the deficiency of dystrophin might not result in the increase in the relevant microRNA in the atrial myocardium of Duchenne muscular dystrophy [62]. Several miRNAs associated with the skeletal muscle development and/or regeneration in the context of Duchenne muscular dystrophy have been intensely investigated, which have revealed several elevated levels of microRNAs including miR-1, miR-133, and/or miR-206 in the serum of Duchenne muscular dystrophy patients [63,64]. Interestingly, dystrophin depletion in the *mdx* mouse may also be associated with a decrease in atrial NOS protein content and/or such microRNAs expression. Compensatory changes in caveolin-1 and NOS may explain the biochemical phenotype of *mdx* atria and could elucidate why Duchenne muscular dystrophy patients might not show an advanced prevalence of the atrial arrhythmias in spite of a decrease in myocardial NOS protein level [65]. The NOS reduction in *mdx* mice might come from failure of the dystrophin complex, which may induce the ubiquitin-mediated degradation of the complex including NOS by the proteasome. In other words, the dystrophin could influence the stability of NOS protein as well as its subcellular localization [61,66]. For example, in vitro studies have described that NOS1 may undertake the proteasomal degradation via the ubiquitination of the calmodulin-association site [67]. As the ubiquitination-proteasomal degradation may specially take place on the inactive and/or damaged form of protein, this autophagy may organize an imperative mechanism for removing nonfunctional NOS proteins [67]. Amplified caveolae domain and NOS expression level may also be linked to the downregulation of miR-124a and/or miR-155 with a greater antioxidant mechanism [68].

The target genes of the downregulated miRNAs might be related to the construction of caveolae, which are supposed to be also indispensable for exosome generation and/or internalization [69]. For example, the relationship among the content of *caveolin-1, caveolin-3*, and hnRNPA2B1, which may play a related role in discerning transport of miRNAs into some exosomal vesicles, has been recognized [70]. In addition, the decrease in *caveolin-3* expression with several miRNAs could also arise the mechanosensitive channel current in myotubes [60,71]. It has been shown that caveolin-3 may be a key for the stabilization and/or trafficking of cardiac ion channels to the membrane [72]. The interaction of miRNAs with their target RNAs may result in either translational inhibition or degradation of the target mRNAs, which can contribute to the suppression of their resultant protein. Interestingly, the muscle-specific ablation of dicer, a key enzyme for the maturation of precursor miRNAs, has shown that miRNAs are also necessary for muscle development [73]. Furthermore, miRNAs have emerged as powerful regulators of skeletal muscle regeneration that might influence many transcriptional pathways [74]. Profiling during myogenic differentiation has shown multiple miRNAs with a distinction of expression patterns in the development of skeletal muscle, which might operate as important myogenic regulators. In particular, some specific miRNAs including miR-1, miR-133 and miR-206 could play imperative roles during muscle cell proliferation, differentiation and/or regeneration [75]. Additionally, miR-3074 can regulate myogenic differentiation by targeting the *caveolin-1* gene, indicating its potential role to progress muscle regeneration [76]. It has been found that exosomes from fibroblasts within muscular dystrophies may exhibit higher levels of miR-199a-5p compared to control exosomes [77]. Exosomes, natural carriers of mRNAs, noncoding RNAs and/or several proteins may vigorously contribute to the cell–cell communication. Interestingly, the injection of fibroblast-derived exosomes from the patients of muscular dystrophies into mouse muscle has shown higher fibrosis rather than those of the control [77]. Therefore, exosomes created from fibroblasts in the muscle of muscular dystrophies could bring a phenotypic alteration of normal fibroblasts to myofibroblasts, enhancing the fibrotic reaction. This transformation may be associated with the conveying of miR-199a-5p and/or decreasing of its target *caveolin-1*, which might suggest a potential therapeutic strategy. The mechanisms of caveolae-mediated cellular uptake might encompass other forms of endocytosis including phagocytosis and/or pinocytosis [78]. Extracellular vesicles may contribute to the pathology of muscular dystrophies, which may become a good biomarker for identifying the status of specific pathological processes that occur in dystrophic muscle [79]. In addition again, the transfer of muscle progenitor cell-derived exosomes might be a favorable approach for treating muscle diseases such as Duchenne muscular dystrophy [80].

## 5. Possible Treatment Tactics with the Alteration of Gut Microbiota against Muscular Dystrophies

The human gut may emerge to be a key organ of bacterium–host communication for the homeostasis of physical health. In addition to its function as a digestive organ, the human gastrointestinal tract is a home of complexed gut microbiota that consists of more than 100 trillion participants [81]. It is well known that gut microbiota may employ various metabolic and/or immunogenic interactions, which could influence many biological activities including enteric protection and/or immune barrier function. Robust cross-talks between gut microbiota and host organs may be an imperative feature of physical homeostasis. Various studies have also emphasized the relationship between gut microbiota and skeletal muscle of muscular dystrophy [82]. In addition, the gut microbiota may play a crucial role in regulating several immune responses, which might influence the pathology of muscular dystrophy. Therefore, the modulation of gut microbiota could contribute to the improvement of muscular dystrophy partly by influencing the expression of genes involved in immune cells-dependent inflammatory reactions [83]. In particular, dysbiosis, known as disruptions of gut microbial composition, may be connected to several immune-mediated and/or autoimmune diseases [84]. In other words, the intricate interactions between the gut microbiota and immune cells are strictly regulated, and dysfunctions in this communication system could lead to several inflammatory conditions [85], which might affect the progression of muscular dystrophy. However, the commensal population comprising the microbiota may extensively diverge among individuals intensely affected by immune responses and/or host genotypes [86]. Additionally, the microbiota is implicated in the development of obesity and diabetes, which could link the gut to systemic insufficient inflammation and/or different muscular adipogenic pathways [87]. Moreover, the quality of bacterial species in the gut microbiota may precisely influence the development of inflammation and/or adiposity in individuals [88]. Interestingly, some studies have correlated the gut microbiota with lifetime cardiovascular risk [20,89]. Short-chain fatty acids (SCFAs) are metabolites made by the gut microbiota with a key role in immune regulation. For example, acetate can enhance the killing activity of macrophages to *Streptococcus pneumoniae* in an NO-dependent manner [90]. In addition, acetate could commonly improve the bactericidal activity of macrophages. Acetate-induced NOS expression and increased NO production has been revealed in endothelial cells, which are also linked to inflammatory cytokine production [91]. These findings may emphasize the requirement to identify strategies targeting the gut microbial system for the treatment of muscular dystrophies. In-depth analyses of the functional relationship between food and microbiota composition may also contribute to the strategy against various human diseases. Possibly, these diseases may be associated with conditions that indorse intestinal dysbiosis with other pathologic circumstances. For instance, an inactive lifestyle has malicious effects on microbial composition, which may also influence treatments of patients with Duchenne muscular dystrophy [92]. Similar to human patients with the muscular dystrophy, *mdx* mice may also exhibit amplified gut peristalsis and relatively decreased fecal excretion [93,94,95,96]. Gut microbiota dysbiosis, characterized by reduced microbial diversity and/or increased maleficent bacteria, has been observed in *mdx* mice. Dysbiosis may cause gut inflammation and immune dysregulation, which can exacerbate the muscle damage of Duchenne muscular dystrophy [97]. Therefore, an improved strategy targeting gut dysbiosis could help to reduce the relevant inflammation and/or could eventually rescue muscle strength [98]. Studies have confirmed the probiotic and anti-gut inflammatory properties in mice fed with high fat diet [99]. Come to think of this, gut microbiota could play a crucial role in connecting food intervention with disease improvement, as the gut could absolutely be influenced by some diets, which should be worth exploring. The potential clinical utility of these diet modifiers would apply into distinct phenotypes of muscular dystrophies in the future. Interestingly, it has directed the clinical heterogeneity of Duchenne muscular dystrophy by stratifying based on the seriousness of muscle dysfunction [100].

## 6. Future Perspectives

**Figure 3:**
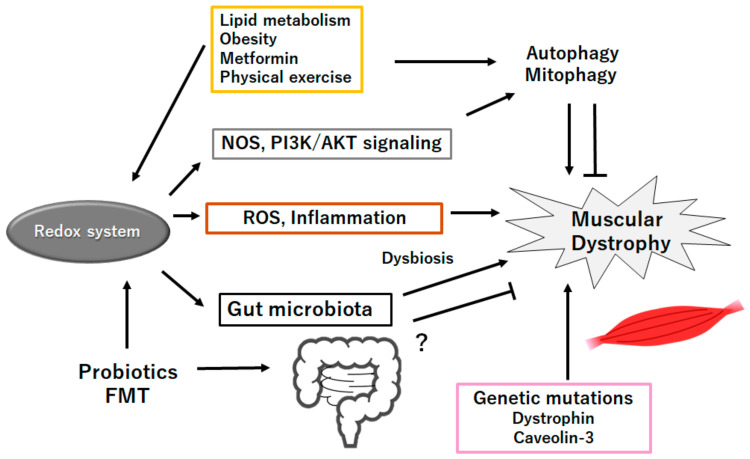
Schematic representation of the probable inhibitory tactics against the pathogenesis of muscular dystrophies. Probiotics and/or fecal microbiota transplantation (FMT) might contribute to the alteration of gut microbiota for the production of SCFAs and/or several miRNAs, which could be beneficial for the treatment of muscular dystrophies. Example factors including metformin as well as exercise known to act on the autophagy signaling are also shown. Note that several important activities such as inflammatory reaction, autophagy initiation, and reactive oxygen species (ROS) production have been omitted for clarity. “?” means for author speculation.

Lipid metabolism disorders and fatty infiltration are typical pathological changes in the muscle of Duchenne muscular dystrophy [101]. In particular, diseased muscle exhibits an excessive accumulation of fibro-adipogenic progenitor cells, which may cause fibrosis and fatty replacement [102]. In addition, the mitochondrial dysfunction caused by a loss of dystrophin may further contribute to lipid deposition in Duchenne muscular dystrophy muscle by impairing glycolipid utilization and enhancing oxidative stress [103]. Abnormal lipid accumulation in muscle might eventually lead to the production of detrimental lipid intermediates [104], which in turn exacerbates mitochondrial damage [105]. This creates a vicious cycle of lipid metabolism in the muscular dystrophy muscle. Lipid metabolism disorder means an abnormal alteration in the lipid profile including hypertriglyceridemia, low-density lipoprotein hypercholesterolemia, and lowered high-density lipoprotein cholesterol in the blood, liver and/or other tissues [106]. Unhealthy lifestyles with high-fat diet, too little exercise, and/or alcohol consumption may be more prone to muscular dystrophy [107]. On the contrary, it can modulate gut–liver interactions to improve lipid metabolism by regulating gut microbiota and their metabolites, including SCFAs [108]. Skeletal muscle and adipose tissue, along with the liver, are the central metabolic organs that are involved in obesity-associated metabolic disorders via the secretion of adipokines, myokines, and/or hepatokines. It is worth mentioning that these metabolic organs may also discharge several exosomes to communicate with peripheral cells along with distant cells/organs and might control body metabolism and/or homeostasis. Again, it has been shown that probiotics and/or fecal microbiota transplantation (FMT) could improve the gut microbiota structure and decrease the amount of harmful bacteria in patients with hyperlipidemia [109]. Alternatively, it is recommended to use probiotics combined with medication to treat patients with muscular dystrophy [109] (Figure 3). To expedite the translation of fundamental knowledge into clinical applications, the search for innovative biomarkers and disease targets gains fruitful consequence. For example, several studies have aimed to identify potential biomarkers for precise assessment in Duchenne muscular dystrophy patients [110]. This endeavor holds the promise of enhancing our understanding and designing innovative therapeutic strategies, ultimately contributing to improved care for individuals struggling with these unbearable diseases.

## 7. Conclusions

Several microRNAs, NO, NOS, and/or caveolins could be involved in the development of certain muscular dystrophies, in which the intricate association between gut and immune/liver/brain/muscle might play an important role. In addition, the correlation between caveolae and exosome might also influence the development of muscular dystrophies. An in-depth knowledge of the role of microRNAs in exosomes may be valuable for advancing new clinical diagnosis and treatment.

## Abbreviations

CAV1	caveolin-1
CAV3	caveolin-3
CNS	central nervous system
DMD	Duchenne muscular dystrophy
FMT	fecal microbiota transplantation
iNOS	inducible nitric oxide synthase
LGMD	Limb–girdle muscular dystrophy
mRNA	messenger RNA
mdx mice	genetic model of Duchenne muscular dystrophy
miRNA	microRNA
miR	miRNA
NO	nitric oxide
NOS	nitric oxide synthase
QOL	quality of life
ROS	reactive oxygen species
SCFAs	short-chain fatty acids
siRNA	short interference RNA
UTR	untranslated region
